# Investigating the feasibility of using transcranial direct current stimulation to enhance fluency in people who stutter

**DOI:** 10.1016/j.bandl.2016.10.003

**Published:** 2017-01

**Authors:** Jennifer Chesters, Kate E. Watkins, Riikka Möttönen

**Affiliations:** Department of Experimental Psychology, South Parks Road, University of Oxford, Oxford, UK

## Abstract

Developmental stuttering is a disorder of speech fluency affecting 1% of the adult population. Long-term reductions in stuttering are difficult for adults to achieve with behavioural therapies. We investigated whether a single session of transcranial direct current stimulation (TDCS) could improve fluency in people who stutter (PWS). In separate sessions, either anodal TDCS (1 mA for 20 min) or sham stimulation was applied over the left inferior frontal cortex while PWS read sentences aloud. Fluency was induced during the stimulation period by using choral speech, that is, participants read in unison with another speaker. Stuttering frequency during sentence reading, paragraph reading and conversation was measured at baseline and at two outcome time points: immediately after the stimulation period and 1 h later. Stuttering was reduced significantly at both outcome time points for the sentence-reading task, presumably due to practice, but not during the paragraph reading or conversation tasks. None of the outcome measures were significantly modulated by anodal TDCS. Although the results of this single-session study showed no significant TDCS-induced improvements in fluency, there were some indications that further research is warranted. We discuss factors that we believe may have obscured the expected positive effects of TDCS on fluency, such as heterogeneity in stuttering severity for the sample and variations across sessions. Consideration of such factors may inform future studies aimed at determining the potential of TDCS in the treatment of developmental stuttering.

## Highlights

• We tested the effect of TDCS and reading practice under temporary induced fluency.
• Fluency on practised and unpractised sentences increased following induced fluency.
• Anodal TDCS relative to sham did not modulate the improvement on sentence reading.
• TDCS reduced stuttering slightly but not significantly for paragraph reading and conversation.
• This feasibility study informs the future application of TDCS in stuttering therapy.

## 1 Introduction

Developmental stuttering is a speech disorder affecting 1% of the adult population. The fluency of speech is interrupted by moments of stuttering, which include repetitions and prolongations of speech sounds, and ‘blocks’ during which speech sounds cannot be produced. Speech therapy for adults who stutter can reduce stuttering symptoms by explicit practice of new speech patterns, such as prolonging phonemes or producing gentle onsets to syllables (Boberg and Kully, 1994, O’Brian et al., 2003, Webster, 1982). However, the benefits do not persist without continued training and practice (Kell et al., 2009, Ward, 2006) making long-term fluency increases difficult to achieve.

People who stutter (PWS) show subtle abnormalities in the structure and function of the brain regions supporting speech. In particular, the inferior frontal cortex (IFC) is consistently highlighted as an affected region. The IFC plays a key role in speech production, comprising regions involved in motor planning as well as integration of sensory signals (Bohland et al., 2010, Guenther, 2006, Hickok and Poeppel, 2007). The first meta-analysis of functional imaging research in developmental stuttering described over-activation of the right IFC as one of three “neural signatures” of stuttering (Brown, Ingham, Ingham, Laird, & Fox, 2005). Two more recent meta-analyses replicated the finding that an over-active right IFC is one marker of the trait of stuttering (Belyk et al., 2015, Budde et al., 2014). Under-activity in the left IFC has been revealed also in several functional imaging studies with PWS (Fox et al., 1996, French et al., 2011, Neumann et al., 2005, Toyomura et al., 2011, Watkins et al., 2008, Wu et al., 1995). Over-activity in the right IFC may compensate for a left hemisphere deficit (Braun et al., 1997, Preibisch et al., 2003). Watkins and colleagues showed that a portion of left IFC – the ventral premotor cortex – was under-active during speaking, and that the white matter underlying this region was disrupted (Watkins et al., 2008). They suggested that this structural deficit affects the integration of sensory and motor information for speech, and the execution of speech motor commands. This hypothesis is in accordance with the results of a meta-analysis of diffusion tensor imaging studies (Neef, Anwander, & Friederici, 2015): white matter integrity is consistently reduced in PWS within the left superior longitudinal fasciculus, including part of the arcuate fasciculus. The affected tracts connect inferior frontal regions (including ventral pre-motor and motor cortex, and IFC pars opercularis) with parietal (inferior parietal lobule, supramarginal and angular gyri), and temporal cortex (superior and middle temporal gyri).

It has been proposed that non-invasive brain stimulation to left IFC could increase speech fluency in PWS by supporting more stable activation of sensori-motor plans within oro-facial primary motor cortex (Neef, Hoang, Neef, Paulus, & Sommer, 2015). The excitability of oro-facial motor cortex is altered in PWS relative to fluent speakers (Neef et al., 2015, Neef et al., 2011). Specifically, the excitability of the tongue motor cortex is enhanced in the left hemisphere during transitions between speech gestures in fluent speakers, but this left-lateralised enhancement of excitability is absent in PWS (Neef, Hoang, et al., 2015). The lack of left-lateralisation of motor activation in PWS is consistent with less efficient communication between the left IFC and motor cortex affecting timely planning of motor sequences. This suggestion is further supported by findings using magnetoencephalography to measure the timing of brain activity during single word reading, which showed that PWS activate left motor cortex prior to left IFC, a reversal of the timing seen in fluent speakers (Salmelin, Schnitzler, Schmitz, & Freund, 2000).

One form of non-invasive brain stimulation that shows promise in the treatment of speech disorders is transcranial direct current stimulation (TDCS). TDCS modulates neuronal excitability by slightly shifting the resting membrane potential of cells (hyper- or de-polarising, depending on current direction). Variation in the placement of the positive (anode) and negative (cathode) electrodes during TDCS affects neuronal excitability and behaviour in different ways, and interacts with other factors such as the duration and intensity of stimulation. For example, placing the anode over the primary motor cortex, and the cathode over the contra-lateral supra-orbital ridge of the forehead increases neuronal excitability in the primary motor cortex (Nitsche & Paulus, 2000). Such stimulation improves motor task performance and learning (Nitsche et al., 2003, Reis et al., 2009). Furthermore, anodal stimulation outside of the motor cortex also has positive effects on targeted behaviours (Holland et al., 2011, Iyer et al., 2005). Critically, the effects of TDCS on behaviour depend upon stimulation being administered in combination with some task that itself engages the targeted brain region in that behaviour. This combination of stimulation and task is key to the promotion of long-lasting behavioural effects (Reis & Fritsch, 2011). TDCS current flow is relatively non-focal, meaning that current is likely to disperse across the targeted region as well as other regions. However, when the target region is activated by a task during TDCS, ongoing plasticity changes in this region can be reinforced by the neuromodulatory effect of TDCS (Bikson et al., 2013, Reis et al., 2015, Stagg et al., 2011).

Studies in healthy participants have shown that speech and language skills can be improved using anodal TDCS to the left IFC. For example, combining anodal left IFC stimulation with a single session of a “tongue-twister” task resulted in increased articulatory skills following the task (Fiori, Cipollari, Caltagirone, & Marangolo, 2014), and anodal left IFC stimulation reduced reaction times during a naming task (Holland et al., 2011). Performance on artificial grammar learning (de Vries et al., 2010) and verbal fluency (Iyer et al., 2005) also improved in healthy people, following anodal left IFC stimulation. Patients with non-fluent aphasia show improved naming ability after TDCS to the left IFC (Baker et al., 2010, Fiori et al., 2010, Monti et al., 2008) and anodal TDCS to the left IFC combined with articulatory training improved speech in patients with acquired apraxia of speech (Marangolo et al., 2011, Marangolo et al., 2013).

The effect of TDCS on developmental disorders of speech and language, including developmental stuttering, has not been investigated to date. A potential concern related to stimulating the malfunctioning speech production system in PWS is that it may increase stuttering. To mitigate this possibility, we decided to apply TDCS concurrently with a temporary fluency enhancer that would promote plasticity in association with fluent speech production. We chose to use choral speech, which involves speaking in unison with another person and induces complete fluency in adults who stutter (Cherry and Sayers, 1956, Kalinowski and Saltuklaroglu, 2003, Kiefte and Armson, 2008, Saltuklaroglu et al., 2009). The effects of choral speech, like other fluency ‘inducers’, are temporary, however, and stuttering typically returns as soon as the second speaker’s voice is withdrawn (Kalinowski & Saltuklaroglu, 2003).

In the current study, we investigated the feasibility of a single-session of anodal TDCS over the left IFC to prolong the temporary fluency induced by choral speech in PWS. The temporary fluency enhancements caused by choral speech also temporarily normalise activity in the left IFC in PWS (Fox et al., 1996, Wu et al., 1995), similarly to the ‘normalisation’ of the speech network shown following a course of fluency therapy (De Nil et al., 2003, Neumann et al., 2005). However, compared to fluency therapy, choral speech gives a relatively effortless, immediate fluency, and does not compromise speech naturalness. We hypothesised that choral speech would induce a ‘fluent mode’ of speech and normalise functioning of the left IFC, and that application of TDCS over the left IFC during this state would promote plasticity associated with speech network activity during fluent speech, e.g. timely communication between left IFC and motor cortex of the articulators. Together these effects would prolong the duration of the ‘fluent mode’ resulting in measureable reductions in stuttering for the TDCS session relative to the sham stimulation session. We predicted that the fluency enhancing effect of choral speech would not persist in the sham session, and that stuttering rates would return to baseline levels once the fluency enhancer was withdrawn.

## 2 Methods

### 2.1 Participants

Sixteen right-handed native English speakers (2 female) took part in the study. All participants were diagnosed with developmental stuttering by a registered Speech and Language Therapist. The mean age of the participants was 30 years (range: 19–58 years). Participants had no history of any communication disorder or neurological impairment, other than developmental stuttering. All participants reported normal hearing and normal (or corrected-to-normal) vision. The Stuttering Severity Instrument, version 3 (SSI-3: Riley, 1994) was used as a standardized measure of stuttering symptoms. The average score across participants on the SSI-3 was 19.4, which is classified as mild (range: 7–28; borderline to moderate stuttering severity). The NRES Committee South Central Oxford C: (11/SC/0482) approved the study. Participants gave their written informed consent, as per the procedure approved by the ethics committee.

### 2.2 Procedure

Each participant completed two experimental sessions that were separated by at least one and no more than two weeks. In each session, they read sentences out loud while listening to another person reading the same sentences. This choral speech practice lasted 20 min and was completed concurrently with anodal TDCS in one session, and sham stimulation in the other. The order of the TDCS and sham sessions was counterbalanced across participants. The experimenter and participant were blind to the stimulation conditions. In each session, speech fluency was assessed prior to the choral speech practice (baseline), immediately following the practice (post-1) and again 1 h later (post-2). Three fluency tasks were used: sentence reading, passage reading and conversation.

### 2.3 Stimulation

In the TDCS session, participants received 20 min of 1 mA stimulation, with the anode placed over left IFC (centred on FC5 according to the 10–20 EEG electrode placement system), and the cathode placed over the right supra-orbital ridge. We used 1 mA stimulation intensity, as previous studies directly comparing stimulation intensities do not show a significant benefit of higher intensity (Boggio et al., 2006, Iyer et al., 2005). In addition, using a higher stimulation intensity study increases the risk of unblinding participants to stimulation condition. A Neuroconn direct current stimulator was used to deliver TDCS. The electrodes measured 5 cm × 7 cm; the anode was placed in portrait orientation (short side horizontal), and the cathode in landscape orientation (long side horizontal). The same electrode placement was used in the sham stimulation session, during which the current was ramped up over 15 s, maintained for 15 s at 1 mA and ramped down over 15 s at the start of the choral speech practice.

### 2.4 Choral speech practice

The sentences used for the choral speech practice and sentence reading task were taken from the IEEE Harvard sentences (IEEE Recommended Practice for Speech Quality Measurements, 1969). Four sets of 24 sentences matched for number of syllables were compiled (each set had an average 10.3 syllables per sentence, and sentences ranged from 10 to 11 syllables). A female native speaker of British English made audio-recordings of two of the four sets; the other two sets were not recorded. The recordings were matched for duration (average in ms (±SD), set 1: 2443 (226), set 2: 2468 (223)), and speech rate (average syllables per second (±SD), set 1: 4.22 (0.33), set 2: 4.19 (0.35)). In each experimental session, one of the two recorded sets was selected randomly for use in the choral speech practice and one unrecorded set was selected that was matched and unpractised for use in the sentence reading task. The set used in the choral speech practice was read aloud solo at baseline, read 10 times during choral speech practice, and read aloud solo again after the practice, at the two outcome time points, as described in Section 2.5. The matched unpractised set was read aloud solo, at baseline and after the choral speech practice, at the two outcome time points, as described in Section 2.5.

The choral speech practice consisted of 10 blocks. Within each block, the 24 sentences from one IEEE set were presented in a random order. Each sentence was displayed on a computer screen for 4.5 s, using Presentation software (Neurobehavioural Systems). The audio recording of the sentence started 1 s after the written sentence appeared. The participant was instructed to start reading the sentence when the audio recording commenced, and to attempt to speak in unison with the recorded voice. A practice run of four trials was used for familiarisation. The familiarisation sentences were not repeated in the training stage.

### 2.5 Speech assessment tasks

At three time points, before the choral speech practice (baseline), immediately after (post-1) and 1 h later (post-2), participants performed three speech assessment tasks in a fixed order; these tasks were performed solo not in chorus. First, they read aloud two sets of 24 sentences from the IEEE (the one used for choral speech practice and a matched unpractised set, as described in Section 2.4). Sentences from the two sets were presented in a random order. The sentences were displayed on a computer screen and the task was self-paced. Second, they read a passage aloud from a printed sheet, and no time constraint was given. Eight passages were used; they were taken from an intermediate ‘English as a Foreign Language’ online training resource (eslfast.com). Passages were matched for number of syllables (average 419, range 360–460). A novel passage was selected at random at each outcome time point. Finally, the experimenter initiated a brief conversation from a set of ten topics. A two-minute sample of conversation at each time point was analysed (average of 335 syllables, range 163–633). Participants’ speech during all tasks was recorded digitally for off-line analysis.

### 2.6 Speech analysis and statistical analysis

All audio-recorded speech samples were transcribed, and stuttered syllables for each sample were counted. These transcriptions were used to calculate the percentage of stuttered syllables (%ss). Stuttered syllables were defined as those containing repetitions of phonemes, prolongation of phonemes or ‘blocks’ (abnormal pauses, often accompanied by audible tension). Other disfluencies, such as interjections or repetitions of multi-syllabic words or phrases, were not counted. The researchers completing these transcriptions were blind to the experimental session. Intra-rater reliability of stutter counts was determined by re-measuring a randomly selected sample of 10% of all recordings. Inter-rater reliability was similarly measured by comparing the stutter counts for two researchers for a randomly selected sample of 10% of all recordings. A strong intra-class correlation was found for both the inter-rater (ICC = 0.97, p < 0.001) and intra-rater (ICC = 0.99, p < 0.001) measurements, indicating high reliability.

The data for all speech assessment tasks showed a significant positive skew, at most time-points. Therefore, a logit transform was used to normalise the data and to allow use of parametric statistics in the analyses. Transformed data were used in all the analyses. We performed a 2 × 3 × 2 repeated measures ANOVA on the sentence reading task, with stimulation (TDCS, Sham), time-point (baseline, post-1, post-2) and sentence set (practised, unpractised) as within-subject factors, to test whether fluency increased (i.e. %ss decreased) following choral speech, and if TDCS modulated any improvement. Simple planned contrasts were used to explore significant main effects of time-point. When a significant interaction between time-point and sentence set was found, we carried out separate 2 × 2 repeated measures ANOVAs, with stimulation (TDCS, Sham) and sentence set (practised, unpractised) as factors, at each time-point.

We performed separate 2 × 3 repeated measures ANOVAs on the paragraph reading and conversation tasks, with stimulation (TDCS, Sham) and time-point (baseline, post-1, post-2) as within-subject factors, to test whether fluency increased following choral speech, and if TDCS modulated any improvement.

## 3 Results

All participants were completely fluent during choral speech. Fluency varied for some individuals between the two baseline sessions (see Table 1), however there were no significant differences between TDCS and sham sessions in the logit transformed or raw %ss scores before stimulation and choral speech practice in any of the tasks. There was also a considerable range of %ss across participants (baseline sentence reading: 0–29%ss, baseline passage reading; 0.2–22.8%ss, baseline conversation: 1.1–11.7%ss). A number of participants produced fluent speech (less than 1%ss) during the baseline measures on one or more tasks in one experimental session. Five participants were fluent in one baseline session during sentence reading, and two of these participants were also fluent in one baseline session during passage reading. However, no participant was fluent on any task in both baseline sessions. For completeness, we present the results of analyses using data from all participants for the sentence and passage reading tasks, and the conversation task (see Table 1), and also include the results of analyses with data excluded from participants who were fluent at baseline, where this revealed additional significant effects (see Table 2: note that this table does not contain data for the conversation task, as no participants were fluent at baseline during this task).

### 3.1 Sentence reading task

**Fig. 1:**
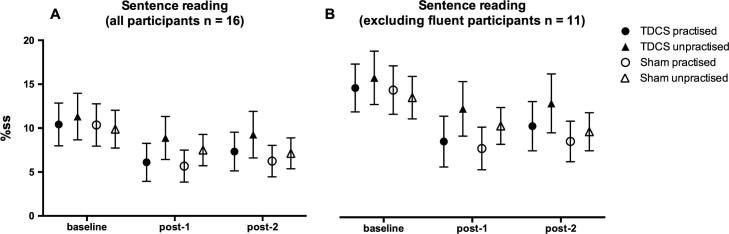
Percentage stuttered syllables (%ss) during sentence reading. %SS scores during sentence reading are plotted before choral speech practice (baseline), immediately following choral speech (post-1) and 1 h following choral speech practice (post-2) for all 16 participants (A), and for 11 participants who produced at least 1%ss in both baseline sessions (B). Symbols show the group mean for the two sentence types (circles – practised, triangles – unpractised) and the two sessions (filled – TDCS, open – sham). Error bars show the standard error of the mean. See text for significant main effects and interactions.

**Fig. 2:**
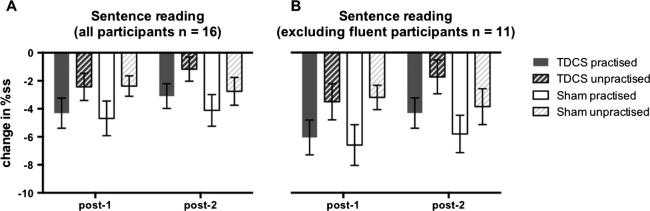
Changes in percentage stuttered syllables (%ss) during sentence reading. Changes in %ss scores from baseline during sentence reading (i.e. baseline score subtracted) are plotted immediately following choral speech practice (post-1) and 1 h following choral speech practice (post-2) for all 16 participants (A), and for 11 participants who produced at least 1%ss in both baseline sessions (B). Bars show the group mean for the two sentence types (solid – practised, striped – unpractised) and the two sessions (grey – TDCS, white – sham). Error bars show the standard error of the mean.

Mean raw and logit transformed %ss scores during the sentence reading task are presented in Table 1 (all participants) and Table 2 (excluding data from participants who were fluent during one session at baseline). When data from all participants were included in the analysis, the results showed that stuttering significantly reduced following the choral speech practice (main effect of time-point: F_2,30_ = 11.19, p < 0.001); this effect was significant immediately after choral speech practice (F_1,15_ = 11.41, p = 0.004, d = 1.81) and was maintained 1 h later (F_1,15_ = 15.34, p = 0.001, d = 2.02), when compared with baseline (Figs. 1A and 2A). The two outcome time points were not significantly different from each other. The choral speech practice significantly reduced stuttering more for the practised compared with the unpractised sentences (interaction between sentence set and time: F_2,30_ = 3.51, p = 0.043; Fig. 1, Fig. 2). Collapsed across all time points, the practised set was spoken more fluently than the unpractised (main effect of sentence set: F_1,15_ = 22.44, p < 0.001, d = 2.41). Analysing each time point separately, there was no significant difference between the sentence sets at baseline. The interaction was due to significantly less stuttering for practised compared with unpractised sentences at the first outcome time point (post-1: F_1,15_ = 13.63, p = 0.002, d = 1.91) but this difference was no longer significant 1 h later (post-2: F_1,15_ = 3.66, p = 0.075, d = 0.98). Importantly, these effects were not modulated by TDCS (there was no main effect of stimulation, or any significant interactions involving stimulation session).

Re-analysis excluding data from the five participants who were fluent during one of the two baseline sessions revealed a very similar pattern of results, with significant differences as reported for the complete data set. For this analysis, however, the difference between practised and unpractised sentence was significant at both outcome time points (F_1,10_ = 12.45, p = 0.005, d = 2.20: Figs. 1B and 2B).

### 3.2 Passage reading task

**Fig. 3:**
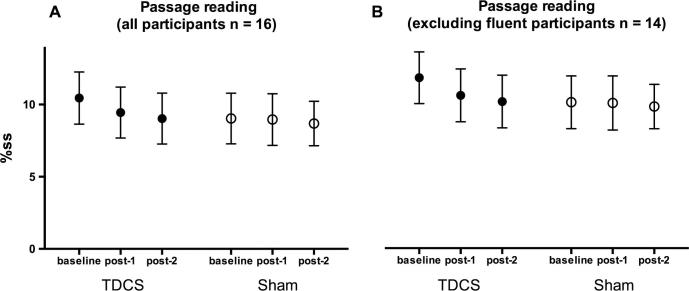
Percentage stuttered syllables (%ss) during passage reading. %ss scores during passage reading are plotted before choral speech practice (baseline), immediately following choral speech practice (post-1) and 1 h following choral speech practice (post-2) for all 16 participants (A), and for 14 participants who produced at least 1%ss in both baseline sessions (B). Symbols show the group mean for the two sessions (filled – TDCS, open – sham). Error bars show the standard error of the mean.

**Fig. 4:**
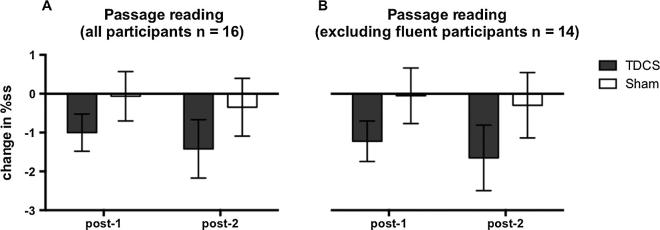
Changes in percentage stuttered syllables (%ss) during passage reading. Changes in %ss scores from baseline during passage reading (i.e. baseline score subtracted) are plotted immediately following choral speech practice (post-1) and 1 h following choral speech practice (post-2) for all 16 participants (A), and for 14 participants who produced at least 1%ss in both baseline sessions (B). Bars show the group mean for the two sessions (grey – TDCS, white – sham). Error bars show the standard error of the mean.

Mean raw and logit transformed %ss scores during the passage reading task are presented in Table 1 (all participants) and Table 2 (excluding data from participants who were fluent during one of the baseline sessions). When data from all participants were included in the analysis, results showed that there was no significant reduction in stuttering during passage reading following choral speech practice (no main effect of time-point). Stuttering did not differ overall between the TDCS and sham sessions (no main effect of stimulation session) and TDCS did not modulate any effects (no significant interaction between time-point and stimulation session) (Figs. 3A and 4A). A second analysis of the data, which excluded participants who were fluent during one baseline session, also did not reveal any significant effects (Figs. 3B and 4B). However, although the sessions were not significantly different, examination of the means (Fig. 3, Fig. 4, Table 2) revealed that there was a greater reduction in stuttering in the TDCS session compared to the sham session at both outcome time points and for both the full data set and the reduced data set. For the TDCS session, the absolute reductions of 1 and 1.4%ss at post 1 and post 2 respectively represent relative decreases of 10% and 14% of the baseline stuttering rate. In contrast, for the sham session, the reductions were less than 0.5%ss and represent decreases of 1% and 4% (post 1 and post 2, respectively) of the baseline.

### 3.3 Conversation task

**Fig. 5:**
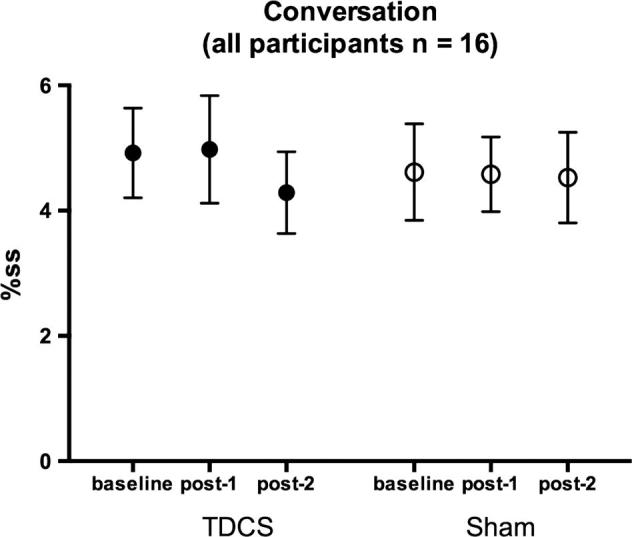
Percentage stuttered syllables (%ss) during conversation. %SS scores during conversation are plotted before choral speech practice (baseline), immediately following choral speech practice (post-1) and 1 h following choral speech practice (post-2) for all 16 participants. No participants were fluent at baseline in this task. Symbols show the group mean for the two sessions (filled – TDCS, open – sham). Error bars show the standard error of the mean.

**Fig. 6:**
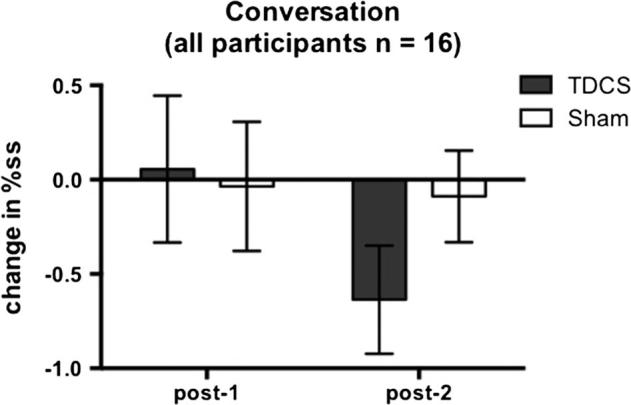
Changes in percentage stuttered syllables (%ss) during conversation. Changes in %ss scores from baseline during conversation (i.e. baseline score subtracted) are plotted immediately following choral speech practice (post-1) and 1 h following choral speech practice (post-2) for all 16 participants. No participants were fluent at baseline in this task. Bars show the group mean for the two sessions (grey – TDCS, white – sham). Error bars show the standard error of the mean.

Mean raw and logit transformed %ss scores during the conversation task are presented in Table 1 (no participants were fluent at baseline on this task). There was no significant reduction in stuttering during passage reading following choral speech practice (no main effect of time-point). Stuttering did not differ overall between the TDCS and sham sessions (no main effect of stimulation session) and TDCS did not modulate any effects of choral speech (no significant interaction between time-point and stimulation session (Fig. 5, Fig. 6). As for the passage reading, although difference between sessions was not significant, examination of the means (Fig. 5, Fig. 6, Table 1) revealed that there was a greater reduction in stuttering following TDCS compared to sham stimulation at 1 h after the practice. For the TDCS session, an absolute reduction of 0.63 in %ss represents a 13% decrease of the baseline stuttering rate. In contrast, for the sham session, the reduction was less than 0.1%ss, representing a 2% decrease of the baseline.

### 3.4 Other measures of speech fluency

Further analyses of other speech measures, such as stuttering rate (the number of stuttered syllables produced per minute) and speaking rate (the total number of syllables produced per minute) showed the same pattern of results as seen for the analysis of %ss.

## 4 Discussion

In this study, we investigated whether a single session of anodal TDCS to the left IFC could enhance fluency in PWS. We temporarily induced fluency during stimulation using choral speech. We hypothesised that when fluent speech was induced and combined with anodal TDCS, some reduction in stuttering would be maintained once the choral speech was withdrawn. As expected, choral speech successfully induced complete fluency in all participants while they received either TDCS or sham stimulation. Speech fluency on sentence reading improved for at least 1 h after the practice ended, but the effect was not significantly modulated by TDCS. For paragraph reading and conversation following TDCS, speech fluency improved slightly relative to the baseline assessments, but these were not significant when compared with the sham session. Below, we discuss the results in detail, the lessons learned from this feasibility study, and future directions.

We found a significant reduction in stuttering during sentence reading following the choral speech practice, in both the TDCS and sham sessions. The stuttering reduction was greater for the sentences that had been used during choral speech practice than for the unpractised sentences. Initially, this result appeared inconsistent with claims based on previous research that describe a lack of persistence in the fluency enhancing effects of choral speech (Kalinowski & Saltuklaroglu, 2003). However, it is likely that the fluency increase observed in the current study following choral speech was induced by the 10 repetitions of the sentences during the practice, rather than the presence of a second voice. Repetition adaptation has been demonstrated previously in people who stutter (Max & Baldwin, 2010), and can persist for at least 24 h. In addition, both the present study and the previous study (Max & Baldwin, 2010) showed reduced stuttering on novel sentences when these were randomly presented amongst practised sentences, but this reduction was smaller than for the practised sentences. This may be due to carry-over, or transfer, of motor adaptation, or to adaptation to the speaking situation. We did not find support for our prediction that combining TDCS with choral speech would result in a greater stuttering reduction relative to the reduction seen in the sham session. We found no significant difference between the TDCS and sham sessions in the size of the reduction in stuttering during sentence reading following choral speech practice. It is possible that any additional effect of TDCS on stuttering was masked by the repetition adaptation effect during sentence reading.

We were also interested in whether the effects of TDCS on fluency might generalize to novel speech tasks not used during the choral speech practice. In the sham session, the size of the reduction in stuttering during passage reading or conversation was negligible. In the TDCS session, there was a larger reduction in stuttering during passage reading and conversation following choral speech but the size of this effect did not differ significantly relative to the sham session (see Fig. 4, Fig. 6). We suggest that this lack of statistical significance was influenced by two sources of variability: inter-individual variability in the response to TDCS, and both inter- and intra-individual variability in stuttering rates.

It is well known that the response to TDCS shows considerable variation among individuals. For example, anodal TDCS to primary motor cortex produces facilitation of cortico-spinal excitability in only approximately three quarters of young, healthy participants (Wiethoff, Hamada, & Rothwell, 2014). Some of the variability seen in the current study may be a consequence of inter-individual variations in the induced current flow and in responsivity to this current. Although TDCS is often used to induce changes in the cortex directly underlying the target electrode, the induced currents are complex, and are influenced by individual brain morphology (Bestmann, de Berker, & Bonaiuto, 2015). Variability is seen in the behavioural, as well as the physiological, response to TDCS and the relationship between effects at these different levels is not well understood. Future TDCS studies with people who stutter might benefit from an individualised approach, for example, by tailoring electrode placement and stimulation intensity to each participant For this approach, a dependent measure such as a robust behavioural effect or measures of cortical excitability might be useful.

A second source of variability in the current study was the inter- and intra-individual variability in stuttering symptoms, which is characteristic of this speech disorder (Karimi et al., 2013, Soderberg, 1962, Yaruss, 1997). Stuttering moments are intermittent, and their severity can vary considerably across time and contexts. We used both reading and conversation samples, in order to gain a representative view of stuttering symptoms. On average, the participants were more fluent in conversation than in the reading tasks, although four participants showed the opposite pattern; so, our group was heterogeneous in this regard. In addition, participants varied in overall stuttering severity, and a number of participants who had milder symptoms produced fluent speech during some of the baseline speech assessment tasks. Excluding these participants from analysis yielded a numerically larger reduction in stuttering on passage reading in the TDCS session compared to the sham session (which showed negligible change), but this difference remained non-significant. This suggests that in order to assess the therapeutic potential of brain stimulation to treat fluency disorders, future studies would benefit from using a more homogeneous sample of people and those with more severe stuttering symptoms, thereby increasing the sensitivity to detect the beneficial effects of TDCS. This does pose some challenges, however, as there is a positive skew in severity in the wider population of people who stutter, with more people having a mild rather than a moderate or severe stuttering severity, at least when measuring core stuttering symptoms (Jones et al., 2006, Soderberg, 1962, Wingate, 2002). Once a therapeutic benefit has been demonstrated in a population of people with at least a moderate severity of stuttering, we can determine the parameters of stimulation that would be effective in the sizeable portion of the population with mild or very mild stuttering severity.

The difference in fluency in conversation and reading tasks across participants highlights another consideration. In this study we measured only primary stuttering characteristics (repetitions of speech sounds, prolongations of speech sounds, and ‘blocks’ in the ongoing flow of speech is involuntarily stopped). However, stuttered speech often includes other (secondary) characteristics such as interjections (“um” “err”), repetitions of larger sections of speech, such as whole phrases, or phrase revisions (Ward, 2008). These characteristics can indicate, in the case of a person who stutters, the avoidance of parts of speech that the speaker predicts may cause a stuttered moment. Such speech strategies occur more commonly during spontaneous speech than during reading and may explain why we found lower levels of stuttering in the conversation task relative to the reading tasks in our study. We included both tasks because reading tasks are more sensitive to stuttering in those PWS who use avoidance to reduce stuttering symptoms, and conversation is the more ecologically valid measure. However, we believe that sensitivity to changes in speech fluency might be improved by measuring both primary stuttering symptoms, and other disfluencies, that could indicate avoidance, and have a negative impact on fluency. An effect of TDCS on primary or secondary stuttering characteristics or both would be a positive outcome.

Sensitivity to changes in fluency may have also been affected by presenting the three speech tasks in fixed order, and by using self-paced reading tasks. PWS generally produce more stuttering when under stress, for example in unpredictable contexts, and speaking under time pressure (Ward, 2008, Wingate, 2002). Randomising task order would also reduce the influence of any one task on those that follow it. Adjustments to these aspects of study design could improve sensitivity in future studies.

A consequence of the intra-individual variation in stuttering was a considerable difference in mean level of stuttering at baseline between the two sessions for the passage reading and conversation tasks. We chose a within-participant design for the study. However, we failed to benefit from the greater sensitivity normally achieved by this design because of the large within-participant variability in baseline stuttering rates. Since the effects of a single session of TDCS are typically small, variance in baseline stuttering is particularly problematic.

Although the effects induced by single TDCS sessions are generally small and relatively short-lived, they increase cumulatively when combined with training over multiple days (Baker et al., 2010, Meinzer et al., 2014, Reis et al., 2009). Therefore, the small decreases in stuttering found here in a single TDCS session might be expected to accumulate over multiple sessions to a greater degree than during sham (or no) stimulation. A future direction for research into the potential benefits of TDCS to people who stutter would be to focus on a multiple session approach; we are currently implementing this approach.

One of the aims of this single-session feasibility study was to rule out the possibility that the application of anodal TDCS during speaking might decrease fluency in PWS, either during or following the stimulation. The results do not indicate that our TDCS protocol had a detrimental effect on speech fluency, which was important to establish before continuing with multiple session studies. Furthermore, the small reductions seen here following choral speech in a single TDCS session (a decrease of around 1.5% stuttered syllables in passage reading) are clinically relevant, considering that a moderate to severe level of stuttering is 15–20% stuttered syllables (Onslow, 2000). This indicates the potential for TDCS to reduce stuttering, which should be explored with further research.

We targeted left IFC as a critical brain region in speech production with anodal TDCS that was expected to enhance neuronal excitability. This region was selected for stimulation because it has a key role in co-ordinating the planning and execution of speech movements, and is typically less active in PWS during speech, than in fluent speakers. For future TDCS studies, we may wish to consider targeting other brain regions implicated in developmental stuttering. The neural signatures suggested by meta-analysis of functional imaging studies included over-activity in right IFC, and cerebellar vermis, and under-activity in the auditory cortex. For example, a feasible alternative approach is to suppress over-activity in the right IFC, which has been an effective intervention in aphasia using cathodal TDCS (Kang, Kim, Sohn, Cohen, & Paik, 2011) and repetitive TMS (Naeser et al., 2011). However, this approach may not be beneficial if the right-hemisphere activation is compensating for a left-hemisphere deficit in stuttering, rather than reflecting maladaptive activity.

Furthermore, TDCS could be combined with different behavioural methods for increasing fluency. We chose to combine TDCS with choral speech, as we aimed to reinforce fluent speech, and choral speech induces fluency immediately and more successfully and to a greater extent than other methods (Saltuklaroglu et al., 2009). Alternative approaches such as altering auditory or other sensory feedback (see Lincoln, Packman, & Onslow, 2006 for review), or providing external rhythmic cueing, could also be investigated. In addition, more traditional speech and language therapy techniques such as ‘fluency shaping’ (Boberg and Kully, 1994, O’Brian et al., 2003, Webster, 1982) could be combined with TDCS. However, these techniques need practice to achieve fluency, and yield more unnatural–sounding speech in the short-term. Consideration of when to apply TDCS within the course of behavioural intervention would be important, in order to avoid the potential for reinforcing disfluent or unnatural sounding speech.

## 5 Conclusions

The current study found that choral speech practice increased speech fluency in PWS on sentence reading, but the size of this increase was not significantly modulated by anodal TDCS to the left IFC. Fluency during two additional speaking tasks – passage reading and conversation – was not significantly increased following temporary fluency enhancement (i.e., choral speech) either with or without anodal TDCS. However, there was a trend towards stuttering reduction in passage reading and conversation, following TDCS coupled with induced fluency, which gives some indication of a positive modulation of fluency that could be stabilised and increased using a multiple session TDCS approach. Stuttering varied considerably among individuals and between sessions and may have obscured the expected positive effects of TDCS on fluency. Lasting fluency is notoriously difficult to achieve for adults with persistent development stuttering. Therefore, the appeal of TDCS to improve therapy outcomes is clear. Even though we found no significant effects of a single session of TDCS on speech fluency in people who stutter, we identified several factors that we believe would usefully inform future research.

## Figures and Tables

**Table 1:** Raw and logit transformed %ss scores for all participants (N = 16).

Fluency assessment task	TDCS session mean %ss (s.e.m.)						Sham session mean %ss (s.e.m.)					
	%ss			Logit transform			%ss			Logit transform		
	Baseline	Post-1	Post-2	Baseline	Post-1	Post-2	Baseline	Post-1	Post-2	Baseline	Post-1	Post-2
*Sentence reading*												
Practised sentences	10.42 (2.43)	6.11 (2.16)	7.33 (2.20)	−2.91 (0.45)	−3.92 (0.49)	−3.26 (0.35)	10.36 (2.41)	5.67 (1.82)	6.24 (1.79)	−2.80 (0.37)	−3.70 (0.41)	−3.48 (0.40)
Unpractised sentences	11.31 (2.65)	8.88 (2.45)	9.25 (2.65)	−2.60 (0.32)	−2.95 (0.34)	−2.94 (0.36)	9.88 (2.15)	7.50 (1.78)	7.12 (1.76)	−2.71 (0.32)	−3.08 (0.33)	−3.22 (0.40)

*Passage reading*												
	10.45 (1.81)	9.45 (1.77)	9.03 (1.76)	−2.28 (0.26)	−2.56 (0.23)	−2.67 (0.26)	9.03 (1.75)	8.96 (1.79)	8.68 (1.54)	−2.74 (0.31)	−2.68 (0.25)	−2.72 (0.27)
Conversation												
	4.92 (0.72)	4.98 (0.86)	4.29 (0.65)	−3.14 (0.17)	−3.15 (0.17)	−3.31 (0.18)	4.62 (0.77)	4.58 (0.59)	4.53 (0.72)	−3.23 (0.17)	−3.17 (0.14)	−3.24 (0.17)

**Table 2:** Raw and logit transformed %ss scores excluding participants who were fluent at baseline for either the sentence reading task (N = 11) or the passage reading (N = 14). No participants were excluded from the conversation task, as none were fluent at baseline.

Fluency assessment task	TDCS session mean %ss (s.e.m.)						Sham session mean %ss (s.e.m.)					
	%ss			Logit transform			%ss			Logit transform		
	Baseline	Post-1	Post-2	Baseline	Post-1	Post-2	Baseline	Post-1	Post-2	Baseline	Post-1	Post-2
*Sentence reading*												
Practised sentences	14.60 (2.70)	8.55 (2.87)	10.29 (2.79)	−1.97 (0.25)	−3.07 (0.44)	−2.53 (0.31)	14.37 (2.74)	7.77 (2.41)	8.56 (2.29)	−2.03 (0.28)	−3.23 (0.54)	−2.83 (0.37)
Unpractised sentences	15.76 (3.01)	12.25 (3.08)	12.87 (3.33)	−1.88 (0.25)	−2.28 (0.29)	−2.20 (0.27)	13.52 (2.40)	10.32 (2.08)	9.66 (2.15)	−2.03 (0.23)	−2.40 (0.25)	−2.48 (0.24)

*Passage reading*												
	11.80 (1.79)	10.57 (1.83)	10.15 (1.82)	−2.17 (0.19)	−2.33 (0.20)	−2.42 (0.22)	10.10 (1.82)	10.05 (1.87)	9.80 (1.54)	−2.45 (0.24)	−2.45 (0.20)	−2.43 (0.20)
